# Extended reality simulator for dynamic visualization and evaluation of stereotactic arrhythmia radioablation (STAR) treatment plans within RAVENTA trial

**DOI:** 10.1093/ehjdh/ztag100

**Published:** 2026-07-05

**Authors:** Domenico Riggio, Joana Leitão, Melanie Grehn, Judit Boda-Heggemann, Adrian Zaman, João Seco, Oliver Blanck, Maria Francesca Spadea

**Affiliations:** Institute of Biomedical Engineering, Karlsruhe Institute of Technology (KIT), Kaiserstraße 12, 76131 Karlsruhe, Germany; Division of Biomedical Physics in Radiation Oncology, German Cancer Research Center (DKFZ), Im Neuenheimer Feld 280, 69120 Heidelberg, Germany; Department of Radiation Oncology, University Medical Center Schleswig-Holstein, Arnold-Heller-Str. 3/Haus L, 24105 Kiel, Schleswig-Holstein, Germany; Department of Radiation Oncology, University Medicine Mannheim, Medical Faculty Mannheim, Heidelberg University, Theodor-Kutzer-Ufer 1–3, 68167 Mannheim, Germany; Department of Internal Medicine III, Cardiology, University Medical Center Schleswig-Holstein, Arnold-Heller-Str. 3/Haus K3, 24105 Kiel, Schleswig-Holstein, Germany; Division of Biomedical Physics in Radiation Oncology, German Cancer Research Center (DKFZ), Im Neuenheimer Feld 280, 69120 Heidelberg, Germany; Department of Physics and Astronomy, Heidelberg University, Albert-Ueberle-Straße 2, 69120 Heidelberg, Germany; Department of Radiation Oncology, University Medical Center Schleswig-Holstein, Arnold-Heller-Str. 3/Haus L, 24105 Kiel, Schleswig-Holstein, Germany; Institute of Biomedical Engineering, Karlsruhe Institute of Technology (KIT), Kaiserstraße 12, 76131 Karlsruhe, Germany

**Keywords:** Extended reality, Stereotactic arrhythmia radioablation, Multidisciplinary therapy planning, Ventricular tachycardia, Multimodal data integration, Mixed reality

## Abstract

Stereotactic arrhythmia radioablation (STAR) represents an emerging non-invasive treatment for therapy-refractory ventricular tachycardia. Yet, planning remains challenged by multimodal cardiac imaging integration, electroanatomical mapping (EAM) transfer, and cardiorespiratory motion effects on dose delivery. Current radiotherapy (RT) planning systems offer mainly static visualization and limited access to intramural myocardial structures, hindering communication between cardiology and radiation oncology teams. We present a novel extended reality (XR) simulator designed to dynamically visualize STAR-relevant imaging and planning data. The system integrates diastolic cardiac CT, respiratory-binned 4DCT, anatomical segmentations, EAM data, and phase-recomputed RT dose distributions within an XR environment. Cardiac structures are propagated across respiratory phases using deformable registration, while dose distributions are recomputed on each respiratory-binned CT, enabling phase-specific inspection of dose conformality for both planning target volumes (PTVs) and cardiac target volumes (CardTVs). The resulting time-resolved volumetric dataset is rendered in XR, allowing clinicians to explore cardiac motion, visualize intramural dose deposition, and jointly assess target and organ-at-risk dynamics. This supports qualitative evaluation of dose-motion interplay and interdisciplinary interpretation of intramural targets. The system was tested on three STAR patients enrolled in the RAVENTA trial. Motion analysis revealed PTV centroid displacement amplitudes over the breathing cycle of up to 17.5, 10.2, and 8.9 mm for patients 1, 2, and 3, respectively, with conformity number variations of 0.38, 0.34, and 0.19. Expert evaluation showed positive perceived utility for target-anatomy-dose understanding, motion interpretation, and multidisciplinary communication. This proof-of-concept demonstrates the feasibility and potential clinical value of XR-based motion-aware dose visualization for STAR planning.

## Graphical Abstract

**Graphical Abstract:**
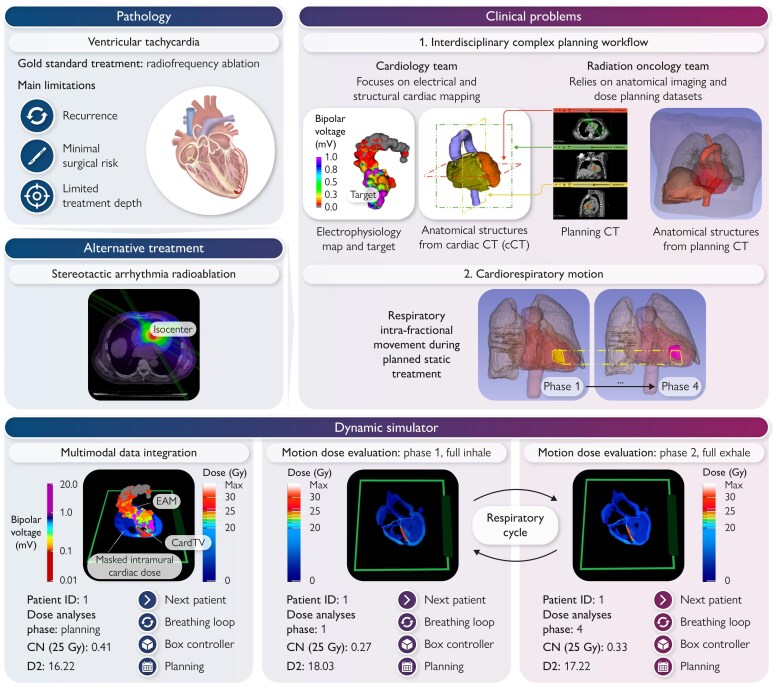

## Introduction

**Figure 1:**
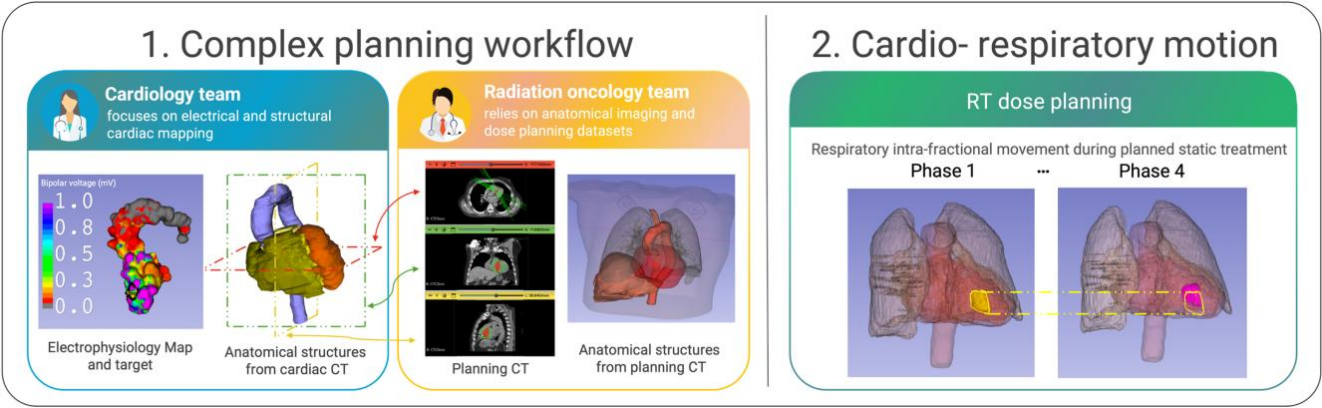
Challenges in STAR treatments. Panel 1, complex planning workflow: STAR requires integration of multimodal data from different clinical teams. The cardiology team defines the ablation target based on EAM and structural information from cardiac CT, while the radiation oncology team relies on anatomical imaging from the planning CT to design the RT dose plan. Integration of these datasets is complicated by differences in imaging modalities and spatial reference frames. Panel 2, cardiorespiratory motion: intrafractional respiratory and cardiac motion can displace both the target and nearby organs at risk (OARs) during treatment delivery, potentially compromising target coverage and increasing exposure to surrounding structures. *Icons from flaticon (https://www.flaticon.com)*

Ventricular tachycardia (VT) is a major cause of cardiovascular death and is treated with anti-arrhythmic drugs and catheter ablation.^1^ However, over 30% of the patients experience VT recurrences after standard treatment.^2^ Stereotactic body radiotherapy, mainly used to treat solid tumours, has recently been adapted for the treatment of VT patients who do not respond to standard treatments or for patients who are ineligible for catheter ablation.^3,4,5,6,7,8^ Stereotactic arrhythmia radioablation (STAR)^4^ makes use of a single radiotherapy (RT) dose of 25 Gy, delivered to the underlying arrhythmogenic substrate. The so-called cardiac target volume (CardTV)^8^ is usually defined from the electroanatomical mapping (EAM) procedures and/or from scar imaging on contrast-enhanced cardiac computed tomography (cCT) or magnetic resonance imaging (MRI).^9^ Early investigations showed short-term safety and efficacy of STAR in small clinical trials^3,5,6^ and the treatment is currently investigated in larger registry projects such as STOPSTORM.eu.^4^ Given the early stage of STAR adoption, tools enabling shared interpretation of target definition, motion effects, and dose delivery may support broader and more consistent implementation. Two major challenges for STAR are:

1. Complex planning workflow: integrating electrical mapping and imaging into RT planning requires coordination between cardiology and radiation oncology teams (*Figure 1*, panel 1).
2. Cardiorespiratory motion: managing motion of the target and nearby critical structures (*Figure 1*, panel 2).

Multiple investigational and quality-assurance methodologies have been proposed for registering the EAM to the radiotherapy planning CT and for taking the CardTV into account within the treatment-planning workflow.^9,10^ In contrast, few techniques have been described for back-projecting delivered RT dose onto the EAM to enable direct visualization of the potential electrophysiological effect of STAR in the myocardial region based on target-definition data.

Furthermore, no method has been reported for displaying the dose while accounting for target motion and the displacement of nearby critical structures. While LV motion is relatively small in VT with structural heart disease (5 mm), respiratory motion can reach several centimetres and significantly impact dose delivery.^11^

To account for breathing motion, an internal target volume can be derived from respiratory binned CT, namely 4DCT. However, this volume often extends into close critical organs at risk (OARs) or intra-cardiac structures which have specific dose limits not to be violated during treatment planning.^12^ Additionally, current RT planning systems allow for visualization of dose distributions over anatomical structures, but do not provide intuitive access to the internal myocardial walls, where most critical arrhythmogenic substrates reside, particularly the interventricular septum, a frequent target in cardiac radioablation. Traditional planning tools do not allow understanding of how the dose maps onto the inner wall of the myocardium, which is particularly important for intramural targets like the septum. Cardiologists and radiation oncologists often lack a shared visual reference for motion–dose interaction in internal cardiac walls.

To address a gap in the workflow of dynamic visualization and evaluation of STAR, a multimodal XR application was developed that integrates 4D thorax imaging, cardiac imaging, phase recomputed RT dose, anatomical segmentations, and EAM into a single, unified visual framework. The primary objective is to facilitate collaboration between cardiology and radiation oncology teams during the planning of STAR procedures, by providing an intuitive, immersive anatomical representation of cardiac motion and its interaction with the therapeutic dose. The novelty of the work centres on:

1. Performing XR visualization of the delivered dose to a moving target as well as moving OARs.
2. Performing XR visualization and quantitative estimation of the dose conformality delivered to the intramural wall of the myocardium, which is particularly important for intramural targets.

This work introduces an XR-based framework for motion-resolved visualization of radiotherapy dose within intramural myocardial structures for STAR planning. The system integrates EAM, 4DCT, and phase-specific dose recalculation into a single immersive environment, facilitating interdisciplinary interpretation of dose–motion interplay.

## State of the art

### XR for 4D cardiovascular visualization

XR technologies are increasingly adopted to enhance spatial understanding of complex anatomy. In the cardiac domain, dynamic imaging data such as 4D/5D CT offer crucial insights into the interaction of respiratory and cardiac motion with pathological substrates. However, this information is often analysed on 2D displays, limiting clinicians’ ability to intuitively grasp spatiotemporal relationships. XR has emerged as a powerful visualization paradigm to overcome these limitations by enabling immersive exploration of patient-specific anatomy and motion. Mena et al. ^13^ demonstrated that time-sequential, patient-specific 3D cardiac models enhance clinical understanding by integrating the spatial detail of 3D anatomy with the dynamic representation of cardiac motion. Their VR-based 4D heart provided clinicians with an intuitive grasp of complex anatomical relationships evolving over time, offering unique insights valuable for congenital heart disease and dynamic planning. In the field of cardiac surgery planning, Napa *et al*. ^14^ demonstrated how VR applications can enhance presurgical understanding of patient anatomy using magnetic resonance imaging and echocardiography, in valvular heart disease. Their usability study emphasized the role of interactive 3D visualization in improving procedural planning and surgeon confidence. Bindschadler et al. ^15^ developed HEARTBEAT4D, an open-source XR framework that allows animated visualization of 4D cardiac CT in Unity^™^, enabling clinicians to interactively explore phase-resolved datasets. This approach facilitates the interpretation of cardiac motion and structural changes throughout the cardiac cycle. Despite these advancements, the development of applications specific to STAR planning remains largely unexplored. Hohmann et al. ^10^ proposed a high-precision CT space target definition method by integrating EAMs into 3D Slicer using a custom plugin, but their workflow remained static and lacked immersive spatiotemporal visualization capabilities.

### XR for RT planning and dose visualization

In radiation oncology, XR applications have shown promise for enhancing RT plan review and communication. Several studies highlight XR for improved spatial comprehension of RT dose distributions, but platforms remain largely static.

Chidambaram *et al*. ^16^ explored the integration of XR systems for stereotactic RT planning, highlighting how immersive visualization of both anatomical and dosimetric information can support interdisciplinary decision making and reduce inter-operator variability. Their work emphasized the role of XR not only in educational contexts but also in improving clinical workflow through intuitive inspection of complex RT data in three dimensions. Similarly, Mages *et al*. ^17^ reported a case report on the use of Microsoft HoloLens^™^ to improve interdisciplinary communication during planning of stereotactic body RT for VT. In this case report, MR visualization helped cardiologists and radiation oncologists collaboratively refine the target definition for a highly challenging case involving the left ventricular summit.

Moreover, while prior XR applications have demonstrated utility in visualizing either 4D cardiac motion or RT dose distributions, none have enabled direct intramural inspection of dose deposition within a beating heart, particularly in anatomically complex and clinically critical regions such as the interventricular septum.

Current planning workflows remain fragmented across disciplines: cardiologists focus on substrate identification using EAM and cardiac imaging, whereas radiation oncologists depend on CT-based tools for RT planning. This disciplinary separation limits the establishment of a shared spatial and functional understanding, especially in cases where motion–dose interplay critically affects both treatment efficacy and safety. These limitations underscore an urgent need for a unified XR-based planning environment that supports multimodal data integration, fosters interdisciplinary collaboration, and enables real time, motion-aware dose evaluation within anatomically accurate cardiac models.

## Materials and methods

### Patient imaging data

This study was conducted using comprehensive retrospective datasets acquired from three patients undergoing STAR of VT within the Radiosurgery for VT (RAVENTA) trial (ethical approval D 555/18, Christian-Albrechts-University of Kiel). For each patient, the dataset comprised the following:

1. Diastolic-phase Cardiac CT (cCT): A contrast-enhanced CT scan, used as the primary anatomical reference for the cardiac chambers;
2. EAM: Exported from the clinical electroanatomical navigation system, containing the acquired electrophysiological point cloud and the ablation target;
3. Respiratory-binned 4DCT: Acquired under free-breathing conditions, with respiratory motion monitored using an external optical tracking system. The data were retrospectively sorted and reconstructed into a discrete set of respiratory phases, capturing the thoracic motion throughout the breathing cycle;
4. RT planning datasets:
  - Planning CT (pCT): A single-phase CT acquired under end-expiration breath-hold conditions and selected as the anatomical reference for dose calculation;
  - RT Struct: a DICOM file containing the Planning Target Volume (PTV) and OARs, delineated by the radiation oncologists;
  - RT Plan: a DICOM file specifying the treatment delivery parameters, including beam geometry;
  - RT Dose: a DICOM file containing the calculated and clinically expected 3D dose distribution, derived from the RT Plan.

For both the cCT and pCT, the corresponding clinical anatomical segmentations were available and were generated manually by clinicians involved in the STAR planning workflow using Varian Medical Systems ARIA RadOnc/Eclipse v13.7.29. These segmentations included the cardiac chambers and the cardiac anatomical reference surface (cSurf) on the cCT, and the corresponding planning cardiac anatomical reference surface (pSurf) on the pCT. Clinical RT structures, including the PTV and OARs, were obtained from the treatment planning dataset and were manually delineated by radiation oncologists using the same clinical treatment planning environment.

Dataset characteristics, relevant to workflow scalability and XR simulation, are summarized in *Table 1*. The number of respiratory phases differed across patients.

**Table 1:** Dataset characteristics relevant to XR simulation

Patient	4DCT phases	4DCT spacing (mm)
Patient 1	8	0.94 × 0.94 × 2.00
Patient 2	8	0.94 × 0.94 × 2.00
Patient 3	11	1.14 × 1.14 × 3.00

The 4DCT spacing represents the image grid resolution of each respiratory phase CT volume.

All data were anonymized prior to analysis, and processing was conducted offline using research software environments.

**Figure 2:**
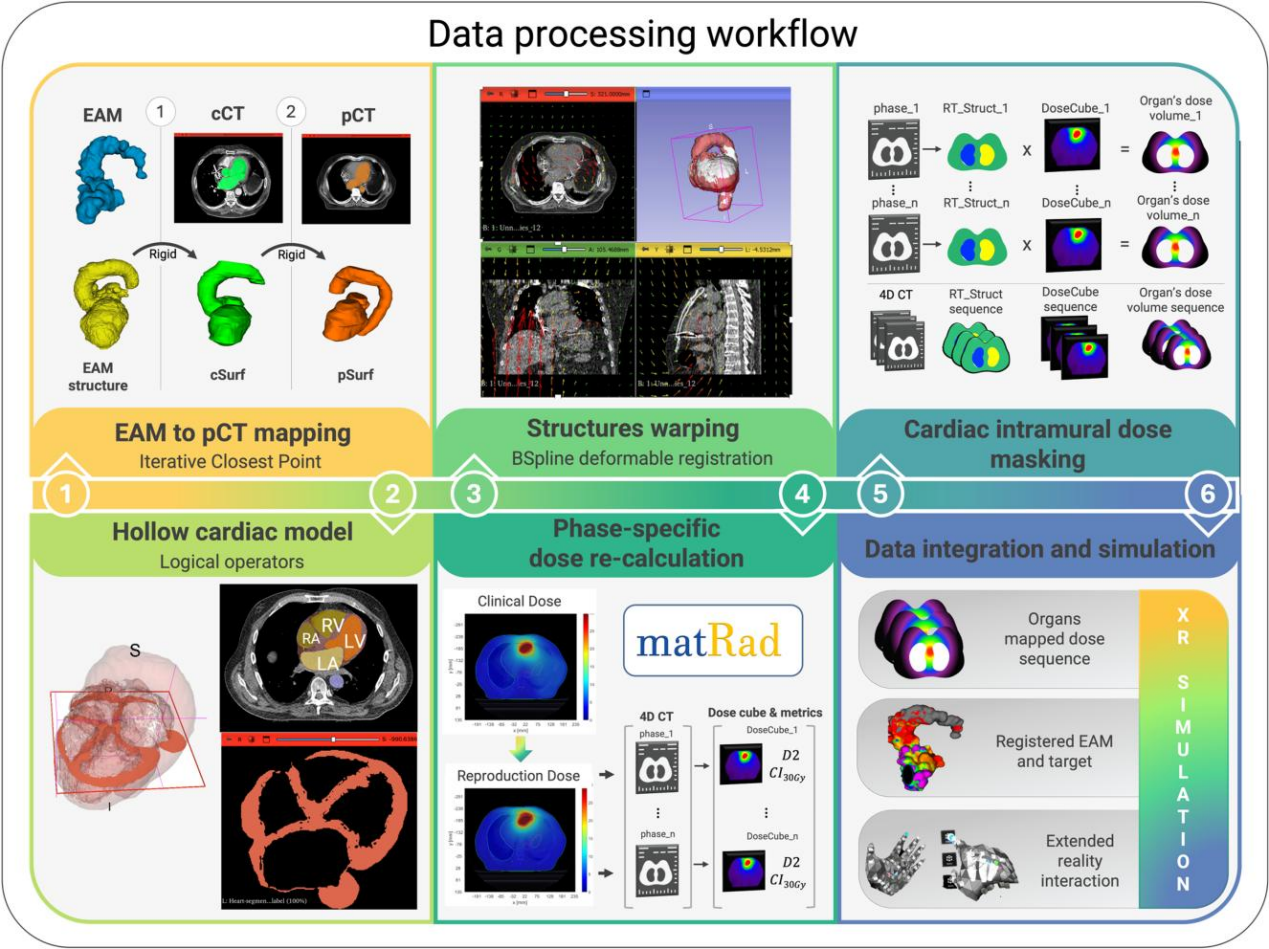
Overview of the six steps data processing workflow used to generate motion-aware simulations for STAR. (1) EAM to pCT mapping: EAM, cCT, and pCT are rigidly registered to align anatomical structures. (2) Hollow cardiac model generation: logical operations are applied to subtract the endocardial blood pools (segmented from the contrast-enhanced CT) from the overall heart volume segmented in the pCT, resulting in a hollow cardiac anatomy model. (3) Structures warping: deformable B-spline registration aligns anatomical structures segmented on the pCT to motion-resolved 4DCT phases. (4) Phase-specific dose recalculation: RT dose is recomputed per 4DCT phase using the open-source matRad platform. (5) Cardiac intramural dose masking: organ specific dose accumulation is computed across the respiratory cycle. (6) Data integration and simulation: registered anatomical structures, dose volumes, and EAM data are combined into an interactive XR environment.

The data processing workflow is depicted in *Figure 2*, where six fundamental steps are illustrated and described in the following paragraphs.

### Data processing workflow

#### Step 1: EAM to pCT mapping

As shown in *Figure 2*, step 1, a sequential automatic surface registration strategy, based on the Iterative Closest Point (ICP)^18^ algorithm with predefined settings, was employed using the Model Registration module of 3D Slicer (v5.6.1)^19,20^ to rigidly align the EAM to the pCT anatomy. It consisted of two stages:

1. Alignment of the EAM to the cSurf, which was segmented by clinicians on the cCT. This reference surface consisted of the left ventricle (LV), left atrium (LA), and ascending aorta. The combined surface served as a stable and reproducible anchor for aligning the EAM with the cardiac anatomy, reducing variability compared with single chamber registrations.
2. Alignment of cSurf to the equivalent anatomical surface pSurf. Also in this case, the segmented structures included LV, LA, and ascending aorta. The output transform was used to map the EAM onto the pCT.

#### Step 2: generation of hollow cardiac morphological model

To enable cardiac intramural dose visualization and analysis, a hollow heart model was generated. The four cardiac chambers were originally segmented by clinicians on the cCT, registered onto the pCT and subtracted from the whole cardiac volume (*Figure 2*, step 2). This Boolean subtraction was performed using 3D Slicer’s Segment Editor module, yielding a solid outer shell representing the myocardial wall.

The resulting hollow cardiac models and intramural masks were independently and visually reviewed by three clinicians. Each reviewer visually inspected the anatomical plausibility of the hollow-heart morphology, the consistency of the chamber subtraction, and the spatial relationship between the myocardial shell, PTV, and surrounding cardiac anatomy. Agreement among the reviewers was reached regarding the suitability of the generated hollow-heart models for qualitative intramural dose visualization and exploratory dose assessment.

**Figure 3:**
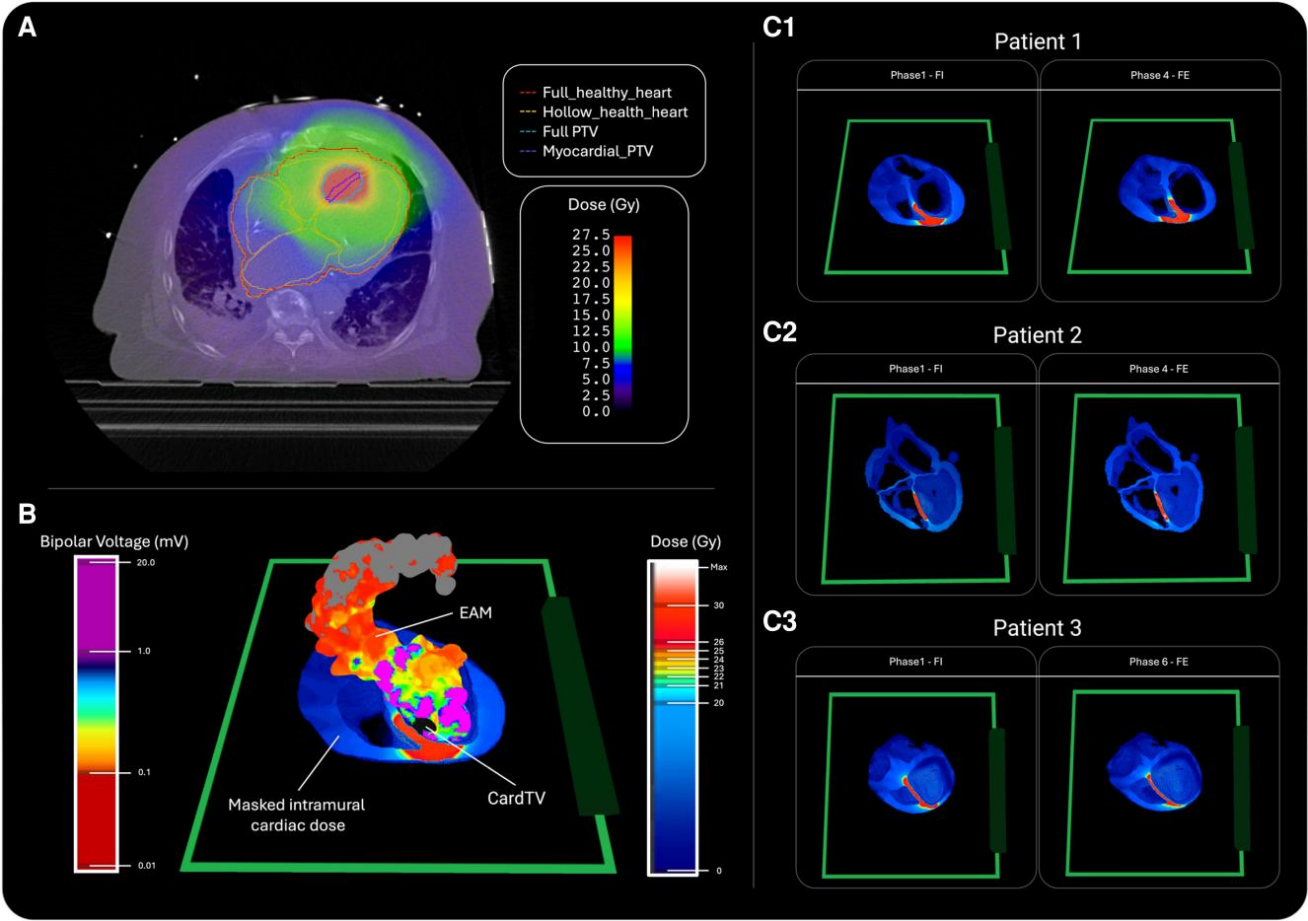
*(A)* Recomputed clinical dose volume overlaid onto the pCT with the considered targets: Full healthy heart, Hollow healthy heart, Full PTV, Myocardial PTV. *(B)* Multidata integration: Masked intramural cardiac dose, EAM and target. *(c.1)* Dose distribution within the inner wall of myocardium in respiratory phase 1, FI, and 4, FE, for patient 1. *(c.2)* Dose distribution within the inner wall of myocardium in respiratory phase 1, FI, and 4, FE, for patient 2. *(c.3)* Dose distribution within the inner wall of myocardium in respiratory phase 1, FI, and 6, FE, for patient 3.

Based on this hollow cardiac model, a myocardial planning target volume (myocardial PTV) was defined to represent the intramural component of the treatment target. This structure was generated as the geometric intersection between the original planning PTV and the hollow cardiac model, thereby restricting the target volume to the myocardial wall. The full healthy heart, hollow healthy heart, full PTV, and myocardial PTV (*Figure 3A*) were subsequently used to evaluate intramural dose coverage and conformality across respiratory phases.

#### Step 3: image registration and structures warping

Using the Sequence Registration module in 3D Slicer, a B-spline deformable registration transform was computed between the pCT (moving volume) and each phase of the 4DCT (fixed volume). The output vector field was employed to warp the PTV, the OARs, and the hollow cardiac model thus generating phase-specific anatomical models adapted to organ motion (*Figure 2*, step 3).

#### Step 4: phase-specific dose recalculation

*Figure 2*, step 4, shows that the clinical dose was initially planned on the pCT and corresponding structures, resulting in a dose distribution that did not account for respiratory motion. To evaluate the impact of such a motion on dose distribution, *matRad*, an open-source MATLAB based RT research toolkit, was used to recompute the dose for each respiratory phase using phase-specific CT geometry.^21^

To ensure consistency across all comparisons, the first step involved reproducing the clinical dose distribution within *matRad* using an equivalent beamlet-based representation, so that all subsequent phase-specific dose recomputations were performed using the same dose engine and calculation settings. For all dose calculations, the dose grid resolution was set to 3 mm × 3 mm × 6 mm. The pCT, structure set, and clinical dose distribution were collected as input. Dose metrics for the target and OARs were extracted from the clinical distribution, including $D_{98}$ and $D_{2}$ for the planning target volume (PTV), and $D_{\min}$, $D_{\mathrm{mean}}$, and $D_{\max}$ for the relevant OARs.^4^

Subsequently, dose was recomputed for each phase of the 4DCT using the same beamlet weights and phase-specific dose influence matrices. The beamlet weights (fluence map) derived from the pCT plan were kept fixed across all phases, without re-optimization. The same dose configuration was applied to all phases without re-optimization. Anatomical structures were propagated to each respiratory phase, as described in step 3, allowing dose evaluation in the context of anatomical variation. This approach enabled a consistent comparison between the planning dose and the phase-specific recomputed dose distributions, isolating the dosimetric impact of respiratory motion on target coverage and OARs sparing.

#### Step 5: cardiac intramural dose masking

To analyse and visualize the dose within the myocardial wall across the respiratory cycle, each phase-specific hollow heart model was converted into an image mask. A voxel-wise multiplication was performed between the hollow heart mask and the corresponding dose volume using 3D Slicer’s Multiply Scalar Volumes (*Figure 2*, step 5). This operation yielded a masked dose volume preserving the hollow morphology and the dose deposited within.

This process was repeated for each respiratory phase and for each patient, resulting in a set of intramural dose maps capturing the dose delivered to the moving heart throughout the breathing cycle. The phase-specific hollow-heart volumes and the resulting cardiac intramural masks were generated in the image space of the corresponding 4DCT phase and therefore retained the voxel spacing and matrix geometry of the respective CT image.

#### Step 6: data integration and simulation

The masked dose volumes, computed for each respiratory phase, were imported into the Unity^®^ engine and processed using the open-source UnityVolumeRendering framework.^22^ The data were concatenated as a time-resolved sequence reproducing the dynamic cardiac motion induced by respiration. The EAM and its associated ablation target, previously co-registered in planning space, were overlaid onto this temporal volume.

An interactive XR interface was developed to enable dynamic exploration of the multimodal dataset, implemented using the Mixed Reality Toolkit^™^ (MRTK) by Microsoft^™^. The head-mounted display (HMD) used for this project was the HoloLens 2^™^. To reduce the computational and memory burden on the headset, the Unity application was executed on a Windows 11 Pro laptop equipped with an Intel Core Ultra 7 255HX CPU, an NVIDIA GeForce RTX 5060 Laptop GPU, and 32 GB RAM, and streamed to the HoloLens 2 using Microsoft Holographic Remoting. In this configuration, volumetric rendering, respiratory phase playback, and interaction logic were processed on the laptop, while the HoloLens 2 was used as the visualization and interaction device. The system enabled toggling of the time sequence, selective visualization of the EAM, and the use of a virtual slicing tool to inspect internal dose distributions within the moving hollow heart geometry (*Figure 2*, step 6).

### Data analysis

#### Quantitative assessment of surface and image registration

The accuracy of surface registration in step 1 was assessed by computing the point-to-surface distances from the transformed EAM to pSurf. Mean surface distance, root-mean-square (RMS) distance, and 95th percentile Hausdorff distance (HD95) were calculated for each patient.

Image registration performed in step 3 was evaluated by computing the Dice Similarity Coefficient (DSC) between the warped OARs and the corresponding OARs manually segmented by a clinician on each 4DCT respiratory phase. OARs included the heart, stomach, and oesophagus, which were selected as clinically relevant structures surrounding the STAR target region and as representative organs with different shapes and deformation characteristics. Results were summarized for each patient and structure as median and interquartile range (IQR) across respiratory phases.

In addition, the physical plausibility of the deformation fields was assessed by inspecting the Jacobian determinant maps generated from the B-spline transforms. This check was used to identify potential nonphysical local deformations, with zero or negative Jacobian determinant values interpreted as indicators of local folding.

#### Dose and motion assessment metrics

In order to assess the conformality of the dose coverage during the breathing cycle, the conformity number was computed as in^23^


(1) $$CN=\left(\frac{{\text{PTV}}_{\mathrm{RI}}}{\text{PTV}}\right)*\left(\frac{{\text{PTV}}_{\mathrm{RI}}}{V_{\mathrm{RI}}}\right),$$

where $PTV_{RI}$ is the PTV receiving the prescribed dose and $V_{RI}$ is the volume of the reference isodose. *CN* was calculated both on the full and myocardial PTV to assess the dose coverage and conformality. D_2_ was computed for the healthy heart (both full and hollow), the oesophagus and the stomach, in order to evaluate high dose spots received by the OARs. Both *CN* and D_2_ were calculated for the structures available in the pCT and in each respiratory phase.

In addition, the Euclidean distances between the centroid of the PTV in each respiratory phase and the centroid of the PTV in phase 1 (corresponding to full-inhale, FI) were computed to quantify the motion amplitude of the target. The Euclidean distance between two centroids $c_{1}=(x_{1},y_{1},z_{1})$ and $c_{2}=(x_{2},y_{2},z_{2})$ was calculated as


(2) $$d=\sqrt{(x_{2}-x_{1}{)}^{2}+(y_{2}-y_{1}{)}^{2}+(z_{2}-z_{1}{)}^{2}}.$$

#### Workflow scalability and computational performance assessment

To provide an estimate of workflow scalability, the approximate processing time required to generate the XR-ready dataset was recorded for the three patients. The timing included data organization, rigid EAM-to-pCT registration, deformable pCT-to-4DCT registration and structure propagation, phase-wise dose recomputation, intramural dose masking, and data export/import into Unity. Clinical contouring time was not included, as the anatomical segmentations and RT structures were already available from the clinical STAR planning workflow. The XR application was executed on a Windows 11 Pro laptop equipped with an Intel Core Ultra 7 255HX CPU, an NVIDIA GeForce RTX 5060 Laptop GPU, and 32 GB RAM, and streamed to the HoloLens 2 using Microsoft Holographic Remoting. The configuration was selected to shift volumetric rendering and respiratory phase playback from the headset to the laptop.

#### Experts’ evaluation

To obtain evidence of perceived clinical utility, a small-scale experts’ evaluation was conducted following development of the XR simulator. Six participants, among cardiologists and radiation oncologists, with clinical or research expertise relevant to STAR planning were invited to interact with the XR system and complete a structured questionnaire. The questionnaire was designed to assess perceived usefulness for STAR treatment planning, with a focus on multimodal data understanding, cardiorespiratory motion interpretation, interdisciplinary communication, ambiguity reduction, and perceived added value compared with the current planning workflow.

Responses were collected using a seven-point Likert scale, where higher scores indicated stronger agreement with the statement. High agreement was defined as a score of 6 or 7.

## Results

### Accuracy of surface and image registration

The mean point-to-surface distance ranged between 2.94 and 3.35 mm among the three patients, with RMS distance included in 4.16–4.37 mm interval and maximum HD95 of 8.86 mm (see *Table 2*). These registration errors were within the millimetric range of the dose grid resolution (3×3×6mm) and comparable to previously reported EAM-to-CT registration accuracy in STAR-related workflows, such as the results reported by Hohmann *et al*. ^10^

**Table 2:** EAM-to-pCT registration accuracy after the two-stage ICP-based surface registration workflow

Patient	Mean (mm)	RMS (mm)	HD95 (mm)
Patient 1	3.35	4.37	8.84
Patient 2	2.94	4.19	8.86
Patient 3	2.95	4.16	8.65

Distances were computed in the final pCT image space as point-to-surface distances from the transformed EAM-derived surface to the pCT-derived reference cardiac surface.

*Table 3* reports the median and IQR of the DSC across the respiratory phases of each patient. Values were >0.80 for oesophagus and >0.85 for heart and stomach. These findings are consistent with previous deformable registration studies in thoracic and abdominal radiotherapy.^24^ In particular, TG-132 reccomandations^25^ indicated clinically acceptable values of DSC the following ranges: 0.85–0.95 for heart, 0.75–0.90 for stomach, and 0.65–0.85 for oesophagus.

**Table 3:** Median (IQR) of the DSC of the OARs

Patient	DSC oesophagus	DSC heart	DSC stomach
Patient 1	0.867 (0.826–0.879)	0.897 (0.875–0.911)	0.890 (0.888–0.895)
Patient 2	0.805 (0.794–0.827)	0.917 (0.902–0.924)	0.886 (0.879–0.895)
Patient 3	0.839 (0.826–0.845)	0.933 (0.919–0.943)	0.965 (0.942–0.987)

Statistics was computed across the breathing phases of each patient.

Inspection of the Jacobian determinant maps did not reveal zero or negative values in the evaluated anatomical regions, indicating no evidence of local folding in the deformation fields used for structure propagation.

### XR visualization of delivered dose

The XR platform enables visualization of:

1. EAM, CardTV, and planned dose distribution (*Figure 3B*).
2. The combined heart anatomical motion and dose distribution variation across the respiratory phases within the inner wall of the myocardium (*Figure 3C.1, C.2, C.3*) respectively for every patient.)

The XR platform, illustrated in the Supplementary material online, supplementary video, enables exploration of all STAR planning data within a single immersive environment. It combines cardiology derived information including the EAM, the CardTV and cardiac anatomy together with radiotherapy data, the planned dose distribution evaluated onto the cardiac volume (*Figure 3B*).

Within this environment, users can interact with the multimodal data through rotation, scaling, and spatial inspection, and are provided with a clipping plane for the masked intramural cardiac dose, facilitating an improved qualitative understanding of the spatial relationships between arrhythmogenic substrates, cardiac anatomy, and dose deposition. The platform allows switching from a static planning view to a dynamic respiratory-cycle visualization. In this mode, the dose distribution is displayed on the hollow myocardial model across the different respiratory phases, enabling phase-resolved inspection of dose variations. Quantitative dose metrics, including D_2_ and CN, are provided for each respiratory phase to support visual findings and enhance the assessment of motion-related dosimetric effects.

### Impact of breathing motion on the estimated dose

**Figure 4:**
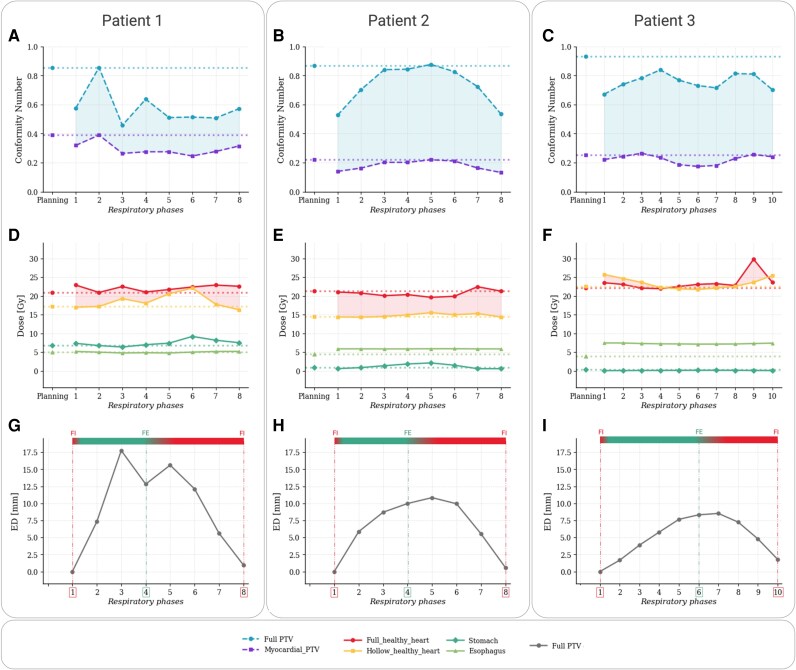
Respiratory-phase analysis of conformity metrics and OAR dose for the three patients included in the study. Panels (*A–C*) report the CN for the full PTV and the myocardial PTV, comparing the planning dose conformity with all respiratory phases. Panels (*D–F*) show the variation of D_2_ for the full healthy heart and hollow healthy heart, stomach, and oesophagus across phases. Panels (*G–I*) display ED of the centroid of the full PTV across the respiratory phases with respect to the first phase centroid of the 4DCT. Shaded regions highlight the Full Inhale (FI) and the Full Exhale (FE) intervals.

*Figure 4* shows the results of the CN for both, full PTV and myocardial PTV, and D_2_ for the standard full healthy heart, the hollow healthy heart, the stomach and the oesophagus, optimized for the pCT and for each respiratory phase. Patient 1 and patient 2 exhibited the largest variation of the CN, ranging from 0.86 to 0.46, and 0.88 and 0.53, respectively.

The CN variation is larger for the full PTV than for the myocardial PTV in all three patients, intuitively because the full PTV has a bigger volume and always encompasses the myocardial PTV. This ensures that the myocardial PTV is always covered, even if not with optimal conformality to the myocardial PTV (as seen represented by an overall lower CN).

The CN represents a measure of how well the prescribed dose conforms to the shape of the target. Referring to Eq. 1, the first term, defined as the volume of the PTV receiving the prescribed dose (PTV_*RI*_) divided by the total PTV volume (PTV), quantifies target coverage. If both volumes are equal, this term is 1; if only half of the PTV receives the prescribed dose, the value is 0.5. The second term is again the volume of the PTV receiving the prescribed dose (PTV_*RI*_) divided by the volume of the patient receiving the prescribed dose (V_*RI*_, which should include the PTV volume), quantifies how the irradiated volume matches the target. If PTV_*RI*_ and V_*RI*_ are equal, this term is 1. When PTV_*RI*_ is small and the V_*RI*_ is large, this term approaches 0; if the opposite is true, the term becomes larger.

When considering all respiratory phases, both PTV volumes remain constant. Furthermore, because dose was recomputed for each phase using the same beamlet weights (fixed fluence), the resulting reference isodose volume (V_*RI*_) remained dosimetrically stable across the 4D cycle. Consequently, the variations observed in *Figure 4* can be attributed to changes in PTV_*RI*_, i.e. the portion of the PTV receiving the prescribed dose. For the full PTV, this allows direct identification of respiratory phases associated with improved target coverage from the corresponding graphs (e.g. phase 2 for patient 1; phases 3–5 for patient 2; and phases 3, 8, and 9 for patient 3).

For the CN of the myocardial PTV, the same logic applies: the PTV volume is fixed and V_*RI*_ remains stable due to the constant fluence, while PTV_*RI*_ fluctuates according to the phase-specific anatomy. However, because the plan was optimized for the full PTV, which is larger than the myocardial PTV, V_*RI*_ will be larger than PTV_*RI*_, so this term will take on a (relatively stable) lower value. Furthermore, if there is variation in the coverage of the full PTV, the myocardial PTV (always contained within the full PTV) will be less sensitive to motion-related changes, as can be seen by comparing the amplitude of CN values for the full and myocardial PTVs. We can therefore also assume that PTV_*RI*_ for the myocardial PTV is relatively stable and close to the myocardial PTV volume itself, so this first term should be close to 1. Consequently, the CN value observed in the graph for the myocardial PTV is effectively representing the second term, the one that quantifies the relationship between the irradiated PTV volume and the total irradiated volume.

As discussed above, lower values of this term indicate that the reference irradiated volume is substantially larger than PTV_*RI*_. Since PTV_*RI*_ for the myocardial PTV is expected to be stable and closely approximate the myocardial PTV volume, the observed overall low CN values for the myocardial PTV indicate that the volume receiving the prescribed dose (V_*RI*_) is considerably larger than the myocardial target. Under the assumption that the myocardial PTV represents the primary therapeutic target, this finding suggests that the current planning strategy irradiates a volume exceeding what would be strictly required for effective intramural target coverage. By using our tool to visualize dose variation across phases and to compare it with the EAM, a cardiologist and a radiation oncologist could jointly redesign the target with smaller margins, potentially improving patient outcomes by avoiding unnecessary irradiation of unaffected regions of the heart. With regard to the OARs, the D_2_ variation is relevant for the hollow healthy heart of patient 1, with a range of 16–22 Gy. In particular, in phase 6, where there is an effect for both the hollow healthy heart and the oesophagus, there could be a clear advantage in allowing a clinician to perform a dedicated visual analysis.

For patient 2, there is a stable difference between the full and the hollow healthy heart, with the hollow healthy heart having a D_2_ that is approximately 5 Gy lower than that of the full healthy heart. As the difference between the full and hollow healthy heart corresponds to including or excluding, respectively, the blood pools in the heart, this means that the clinically relevant heart D_2_ is in practice D_2_ of the hollow healthy heart. Since the heart D_2_, with a maximum constraint of 30 Gy, represents one of the primary dose-limiting factors in STAR treatment planning,^12^ the use of a planning heart D_2_ value of 21 Gy instead of 14.5 Gy suggests that the optimization process may be driven by an overestimated cardiac dose. When D_2_ of the hollow healthy heart is instead considered (approximately 15 Gy), alternative planning strategies could be explored, including potential dose escalation,^26^ while maintaining compliance with cardiac dose constraints. Such an approach may improve target coverage without proportionally increasing irradiation of non-involved myocardial tissue.

For patient 3, the stomach was located very far from the target, resulting in negligible dose. For the heart (empty and full) and the oesophagus, however, the baseline pCT dose does not appear to match or even approximate the recomputed phase-specific dose distributions. The heart D_2_ is above the planned heart D_2_ for almost all phases and exceeds the heart D_2_ constraint (23 Gy) for the full heart in phases 1, 2, and 7–9, and for the hollow healthy heart in phases 1–3, 9–10. Patient 3 also receives a higher dose in the hollow healthy heart, with a median ± interquartile range of 23±2 Gy. No major deviations were found for the stomach and the oesophagus.

### Workflow scalability and computational performance

The workflow was applied to three patient datasets, including 8 respiratory 4DCT phases for Patients 1 and 2 and 11 respiratory phases for Patient 3. Dataset characteristics are reported in *Table 1*. The phase-specific hollow-heart volumes and cardiac intramural masks used for XR visualization were generated in the image space of the corresponding respiratory phase-specific 4DCT volumes; therefore, their resolution matched the respective 4DCT spacing.

The end-to-end processing time required to generate an XR-ready dataset was approximately 2–3 h per patient, excluding clinical contouring and troubleshooting. This estimate included data organization, rigid EAM-to-pCT registration, deformable pCT-to-4DCT registration and structure propagation, phase-wise dose recomputation, intramural dose masking, and data export/import into Unity. The main time-consuming steps were deformable pCT-to-4DCT registration and structure propagation, phase-wise dose recomputation, intramural dose masking, and preparation of the phase-resolved volumetric data for Unity visualization.

During representative interactions, including rotation, scaling, virtual slicing, and respiratory phase playback, the remoted XR application maintained interactive frame rates, typically around 50 FPS and above 40 FPS in most conditions.

This timing and performance assessment should be interpreted in the context of the exploratory nature of the prototype. The current implementation was optimized to assess feasibility, perceived clinical usefulness, and potential impact on interdisciplinary STAR planning, rather than to minimize processing time for routine clinical deployment.

### Experts’ evaluation

**Figure 5:**
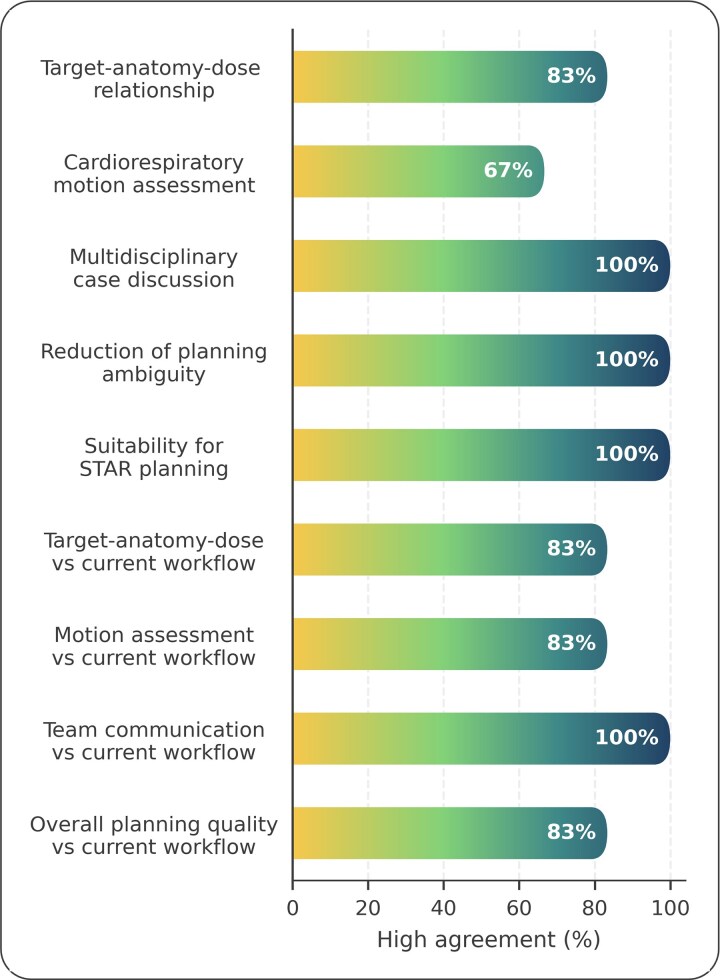
Experts’ evaluation of the XR simulator for STAR planning. Bars show the proportion of high-agreement responses for each questionnaire item, defined as scores of 6 or 7 on a 7-point Likert scale. Percentages indicate the proportion of respondents reporting high agreement. Given the sample size of six respondents, 83%, 67%, and 100% correspond to 5/6, 4/6, and 6/6 respondents, respectively.

Six participants completed the structured questionnaire after evaluating the XR simulator. Overall, the perceived clinical utility of the platform was rated positively across all evaluated domains (*Figure 5*).

High-agreement responses were observed in most evaluated domains. Five of six participants reported high agreement for improved target-anatomy-dose understanding, target-anatomy-dose interpretation compared with the current workflow, motion assessment compared with the current workflow, and overall planning quality compared with the current workflow. Four of six participants reported high agreement for cardiorespiratory motion assessment. All participants reported high agreement for multidisciplinary case discussion, reduction of planning ambiguity, suitability for STAR planning, and team communication compared with the current workflow.

All participants indicated that, for complex STAR cases, they would prefer using the XR simulator in combination with the current planning workflow.

## Discussion

### Principal findings

This work introduces a novel XR simulation platform for STAR, integrating and visualizing patient-specific RT dose evaluated on heart tissues across all respiratory phases of a 4DCT acquisition. This phase-resolved visualization enables direct exploration of how respiratory motion affects both the target volume and surrounding cardiac and extracardiac structures, a key consideration for accurate dose delivery in STAR procedures.

Beyond technical integration, the platform was designed to foster interdisciplinary collaboration between cardiologists and radiation oncologists. By combining EAM, cardiac anatomical segmentations, 4D imaging, and time-resolved dose distributions within a single immersive environment, the system offers the multidisciplinary team a shared view of the case. This shared visual context is particularly valuable when evaluating intramural targets such as the interventricular septum, where the interaction between motion and dose is complex and not easily interpreted through conventional 2D planning tools.

While this is a proof of concept study involving only a small cohort, the approach demonstrates how XR can be leveraged to both enhance spatial understanding and bridge communication gaps in advanced cardiac RT planning. Future work will focus on automating processing, refining motion modelling, integrating clinical feedback, including patient-specific margin assessment, and supporting decisions such as gating or breath-hold when motion substantially affects target coverage or OAR dose.

Beyond technical feasibility, the proposed XR platform has direct implications for clinical decision-making in STAR planning. By enabling phase-resolved visualization of dose deposition within intramural myocardial structures, the system provides clinicians with intuitive access to motion–dose interplay that is not readily interpretable using conventional treatment planning systems.

Phase-resolved visualization of intramural dose deposition may support margin refinement and motion mitigation strategies in selected patients. Identification of respiratory phases associated with target undercoverage or excessive dose to critical structures can inform decisions regarding gated or breath-hold approaches. Additionally, comparison between full and myocardial PTV conformality enables assessment of whether ITV-based strategies adequately reflect intramural motion envelopes. Intramural dose masking further provides clinically meaningful insight into myocardial dose sparing, supporting balanced decisions between treatment efficacy and cardiac safety.

Beyond individual case planning, immersive XR visualization may also support knowledge transfer and interdisciplinary alignment in centres initiating STAR programmes by providing an intuitive representation of complex multimodal data and motion-related dose effects that are difficult to convey using conventional planning tools. The experts’ evaluation supports this perceived clinical value. Participants rated the XR simulator positively for multidisciplinary case discussion, reduction of planning ambiguity, suitability for STAR planning, and perceived added value compared with the current workflow. Target–anatomy–dose understanding was also rated favourably.

The standalone item on cardiorespiratory motion assessment showed comparatively lower agreement, with high agreement in four of six participants. This result should be interpreted in light of the fact that the current implementation visualizes motion derived from respiratory-gated 4DCT data and therefore primarily represents respiratory motion, while cardiac contraction and other sources of uncertainty are not yet explicitly modeled. Moreover, the perceived value of motion visualization depends strongly on the availability, quality, and clinical interpretation of the underlying 4DCT data. Nevertheless, when participants were asked to compare motion assessment with the current workflow, high agreement increased to five of six participants. This indicates that, even though the current implementation focuses mainly on respiratory motion, it may still provide added value over conventional STAR planning discussions, where motion information is generally not integrated as an interactive and explicit component of case review.

Importantly, all participants preferred the XR simulator as a complementary tool to the current planning workflow rather than as a replacement. This supports the interpretation that the main near-term role of the platform is to facilitate shared interpretation, case discussion, and qualitative review of complex STAR plans, while conventional treatment planning systems remain essential for clinical dose calculation, plan optimization, and formal approval.

### Challenges and limitations

As with any proof-of-concept implementation involving multimodal clinical data, the proposed XR simulator presents technical and workflow-related aspects that should be considered when interpreting the results. One relevant aspect concerns the computational requirements associated with high-resolution volumetric CT data, phase-resolved dose distributions, and time-sequential visualization across multiple respiratory phases. In the present implementation, this was addressed by using Microsoft Holographic Remoting, which shifted volumetric rendering, respiratory phase playback, and interaction processing from the HoloLens 2 to a Windows laptop. This configuration enabled interactive visualization during the proof-of-concept evaluation, while preserving the HoloLens 2 as the visualization and interaction device. As VR/XR hardware continues to improve rapidly, with increasingly powerful standalone and hybrid devices becoming available, we expect computational constraints to become less limiting for similar applications in the near future.

The current preprocessing pipeline required approximately 2–3 h per patient, excluding clinical contouring and troubleshooting. This processing time was acceptable for the present feasibility study, which focused on multimodal data integration, perceived clinical usefulness, and interdisciplinary planning support. The most time-consuming steps were deformable registration for structure propagation, phase-wise dose recomputation using phase-specific dose influence matrices and fixed beamlet weights, intramural dose masking, and data preparation for Unity visualization. Further automation of these steps will be important to improve scalability and facilitate future clinical translation.

Another relevant aspect is the integration of EAM data, which are often stored in proprietary and non-standard formats. In this study, the EAM-to-pCT mapping was performed using a two-stage ICP-based registration with predefined settings and did not require manual landmark placement or manual refinement after input preparation. Nevertheless, the quality of the final alignment depends on the anatomical surfaces used as input, which are derived from clinical segmentations and preprocessing. Therefore, segmentation quality control and verification of anatomical consistency remain important components of the workflow.

Finally, integration of XR-based simulation into routine STAR planning will require dedicated workflows for data transfer, preprocessing, model preparation, and device operation. In our preliminary experience, this was feasible through close coordination between imaging specialists, electrophysiologists, medical physicists, and radiation oncologists. Future work will focus on increasing automation, improving robustness, and standardizing the processing pipeline to facilitate broader use in multidisciplinary STAR planning.

Ongoing advances in segmentation, motion modelling, and XR hardware may facilitate broader clinical integration.

### Future steps

Many of the current limitations in scaling and validating our XR platform also reveal promising opportunities for future development and clinical translation. Having demonstrated proof of concept across three representative STAR cases, future work will aim to conduct comparative studies assessing the added value of this immersive dynamic 4D visualization approach relative to conventional planning tools such as 3D CT, cardiac MRI, and standard dose viewers used in current clinical workflows. A key focus will be to evaluate whether the platform enhances target delineation accuracy, interdisciplinary agreement, and planning efficiency, particularly in complex cases involving intramural substrates and motion sensitive regions.

In parallel, the increasing adoption of remote and hybrid MDT workflows in the post-pandemic healthcare landscape highlights a timely opportunity for XR technologies to support tele-immersive case discussions, enabling distributed teams to interact with multimodal patient data in a shared virtual space.

From a technical standpoint, future efforts will focus on enhancing automation in the processing pipeline, particularly for motion-resolved for phase-specific dose recomputation, segmentation, and data registration.

The convergence of XR, dynamic imaging, and intelligent data integration has the potential to redefine the planning and communication paradigms in this emerging treatment modality. Our future work will continue to explore these intersections with the ultimate goal of improving precision, collaboration, and clinical outcomes in STAR.

## Supplementary Material

ztag100_Supplementary_Data

## Data Availability

The data underlying this article cannot be shared publicly due to ethical and privacy restrictions. Additional information will be provided by the corresponding author upon reasonable request.

## References

[1] Zeppenfeld K, Tfelt-Hansen J, de Riva M, Winkel BG, Behr ER, Blom NA, et al 2022 ESC guidelines for the management of patients with ventricular arrhythmias and the prevention of sudden cardiac death. Eur Heart J 2022;43:3997–4126. doi:10.1093/eurheartj/ehac262 36017572

[2] Ravi V, Poudyal A, Khanal S, Khalil C, Vij A, Sanders D, et al A systematic review and meta-analysis comparing radiofrequency catheter ablation with medical therapy for ventricular tachycardia in patients with ischemic and non-ischemic cardiomyopathies. J Interv Card Electrophysiol 2023;66:161–175. doi:10.1007/s10840-022-01287-w 35759160

[3] Cuculich PS, Schill MR, Kashani R, Mutic S, Lang A, Cooper D, et al Noninvasive cardiac radiation for ablation of ventricular tachycardia. N Engl J Med 2017;377:2325–2336. doi:10.1056/NEJMoa1613773 29236642 PMC5764179

[4] Grehn M, Mandija S, Miszczyk M, Krug D, Tomasik B, Stickney KE, et al Stereotactic arrhythmia radioablation (star): the standardized treatment and outcome platform for stereotactic therapy of re-entrant tachycardia by a multidisciplinary consortium (STOPSTORM.eu) and review of current patterns of star practice in Europe. Europace 2023;25:1284–1295. doi:10.1093/europace/euac238 36879464 PMC10105846

[5] Krug D, Zaman A, Eidinger L, Grehn M, Boda-Heggemann J, Rudic B, et al Radiosurgery for ventricular tachycardia (raventa): interim analysis of a multicenter multiplatform feasibility trial. Strahlenther Onkol 2023;199:621–630. doi:10.1007/s00066-023-02091-9 37285038 PMC10245341

[6] Miszczyk M, Hoeksema WF, Kuna K, Blamek S, Cuculich PS, Grehn M, et al Stereotactic arrhythmia radioablation (star)—a systematic review and meta-analysis of prospective trials on behalf of the STOPSTORM.eu consortium. Heart Rhythm 2025;22:80–89. doi:10.1016/j.hrthm.2024.07.029 39032525

[7] van der Ree MH, Dieleman EMT, Visser J, Planken RN, Boekholdt SM, de Bruin-Bon RHA, et al Non-invasive stereotactic arrhythmia radiotherapy for ventricular tachycardia: results of the prospective STARNL-1 trial. Europace 2023;25:1015–1024. doi:10.1093/europace/euad020 36746553 PMC10062344

[8] Zeppenfeld K, Rademaker R, Al-Ahmad A, Carbucicchio C, De Chillou C, Cvek J, et al Patient selection, ventricular tachycardia substrate delineation and data transfer for stereotactic arrhythmia radioablation: a clinical consensus statement of the European Heart Rhythm Association (EHRA) of the ESC and the Heart Rhythm Society (HRS). Europace 2025;27:euae214. doi:10.1093/europace/euae214. 39177652 PMC12041921

[9] ter Bekke R, Hohmann S, Xie J, Grehn M, Verhoeven K, Volders P, et al Transfer of arrhythmia substrate targets from the cardiac electroanatomical and imaging modalities to the planning computed tomography scan for stereotactic arrhythmia radioablation for refractory ventricular tachycardia—a state-of-the-art review on software developments on behalf of the STOPSTORM.eu consortium. Radiother Oncol 2025;210, 111004, doi:10.1016/j.radonc.2025.111004. 40582572

[10] Hohmann S, Henkenberens C, Zormpas C, Christiansen H, Bauersachs J, Duncker D, et al A novel open-source software-based high-precision workflow for target definition in cardiac radioablation. J Cardiovasc Electrophysiol 2020;31:2689–2695. doi:10.1111/jce.14660 32648343

[11] Stevens RRF, Hazelaar C, Fast MF, Mandija S, Grehn M, Cvek J, et al Stereotactic arrhythmia radioablation (star): assessment of cardiac and respiratory heart motion in ventricular tachycardia patients—a STOPSTORM.eu consortium review. Radiother Oncol 2023;188:109844. doi:10.1016/j.radonc.2023.109844 37543057

[12] Blanck O, Buergy D, Vens M, Eidinger L, Zaman A, Krug D, et al Radiosurgery for ventricular tachycardia: preclinical and clinical evidence and study design for a German multi-center multi-platform feasibility trial (raventa). Clin Res Cardiol 2020;109:1319–1332. 32306083 10.1007/s00392-020-01650-9 PMC7588361

[13] Mena K, Urbain K, Fahey K, Bramlet M. Exploration of time sequential, patient specific 3d heart unlocks clinical understanding. 3D Print Med 2018;4:15. doi:10.1186/s41205-018-0034-7 30649656 PMC6283805

[14] Napa S, Moore M, Bardyn T. Advancing cardiac surgery case planning and case review conferences using virtual reality in medical libraries: evaluation of the usability of two virtual reality apps. JMIR Hum Factors 2019;6:e12008. doi:10.2196/12008 30664469 PMC6352013

[15] Bindschadler M, Buddhe S, Ferguson M, Jones T, Friedman S, Otto R. HEARTBEAT4D: an open-source toolbox for turning 4D cardiac CT into VR/AR. J Digit Imaging 2022;35:1759–1767. doi:10.1007/s10278-022-00659-y 35614275 PMC9712868

[16] Chidambaram S, Palumbo M, Stifano V, McKenna J, Redaelli A, Olivi A, et al The potential for using extended reality technology in interdisciplinary case discussions and case planning in stereotactic radiosurgery: proof-of-concept usability study. JMIR Neurotechnol 2022;1:e36960. doi:10.2196/36960

[17] Mages C, Steinfurt J, Rahm A-K, Thomas D, Majidi R, Kehrle F, et al Recurrent ventricular tachycardia originating from the “lv summit” effectively eliminated by stereotactic irradiation—a case report. HeartRhythm Case Rep 2023;9:802–807. doi:10.1016/j.hrcr.2023.08.009. 38023678 PMC10667122

[18] Besl PJ, McKay ND. Method for registration of 3-d shapes. In: Sensor fusion IV: control paradigms and data structures. Vol. 1611. Spie; 1992, p586–606.

[19] Fedorov A, Beichel R, Kalpathy-Cramer J, Finet J, Fillion-Robin J, Pujol S, et al 3D slicer as an image computing platform for the quantitative imaging network. Magn Reson Imaging 2012;30:1323–1341. doi:10.1016/j.mri.2012.05.001 22770690 PMC3466397

[20] Kikinis R, Pieper S, Vosburgh K. 3D slicer: a platform for subject-specific image analysis, visualization, and clinical support. Vol. 3. New York, NY: Springer; 2014. p277–289. doi:10.1007/978-1-4614-7657-3_19.

[21] Wieser H, Cisternas E, Wahl N, Ulrich S, Stadler A, Mescher H, et al Development of the open-source dose calculation and optimization toolkit matRad. Med Phys 2017;44:2556–2568. doi:10.1002/mp.12251 28370020

[22] Lavik M . Unityvolumerendering: Volume rendering in unity. https://github.com/mlavik1/UnityVolumeRendering, 2023. https://github.com/mlavik1/UnityVolumeRendering. Accessed: 2025-11-24.

[23] Van’t Riet A, Mak AC, Moerland MA, Elders LH, Van Der Zee W. A conformation number to quantify the degree of conformality in brachytherapy and external beam irradiation: application to the prostate. Int J Radiat Oncol Biol Phys 1997;37:731–736. 9112473 10.1016/s0360-3016(96)00601-3

[24] Nenoff L, Amstutz F, Murr M, Archibald-Heeren B, Fusella M, Hussein M, et al Review and recommendations on deformable image registration uncertainties for radiotherapy applications. Phys Med Biol 2023;68:24TR01. doi:10.1088/1361-6560/ad0d8a PMC10725576 37972540

[25] Guy CL, Weiss E, Che S, Jan N, Zhao S, Rosu-Bubulac M. Evaluation of image registration accuracy for tumor and organs at risk in the thorax for compliance with tg 132 recommendations. Adv Radiat Oncol 2019;4:177–185. doi:10.1016/j.adro.2018.08.023 30706026 PMC6349597

[26] Kovacs B, Mayinger M, Ehrbar S, Fesslmeier D, Ahmadsei M, Sazgary L, et al Dose escalation for stereotactic arrhythmia radioablation of recurrent ventricular tachyarrhythmia—a phase II clinical trial. Radiat Oncol 2023;18. doi:10.1186/s13014-023-02361-x PMC10634182 37941012

